# On Disorders of the Cerebral Circulation, and on the Connexion between Affections of the Brain and Diseases of the Heart

**Published:** 1846-10

**Authors:** 


### Art. IX.

*On Disorders of the Cerebral Circulation, and on the Connexion between Affections of the Brain and Diseases of the Heart.* By George Burrows, m.d., late Fellow of Caius College, Cambridge, Fellow of the Royal College of Physicians, London, Physician and Lecturer on the Principles and Practice of Medicine at St. Bartholomew's Hospital.—*London,* 1846. 8vo, pp. 220.

The sensations experienced by all persons on placing the head in a dependent position, or on otherwise favoring congestion of the cerebral vascular system, are so determinate, that it has been almost universally received as a fixed principle, that the amount of blood within the cranium may be increased. And yet it cannot be denied, that an opposite opinion has been broached and advocated, for it seems to be a general law in metaphysics, that every principle, whether true or false, shall have its antagonistic principle. In the present work, Dr. Burrows aims at a twofold object: he proposes to show, firstly, that this opposite opinion is erroneous; and, secondly, that changes in the circulation within the cranium have a much wider pathological scope than they are generally supposed to possess.

The doctrine that the quantity of blood circulating within the cranium is, under all circumstances, a fixed quantity, is an offset from the iatro-mathematical school of Borelli, and was promulgated by Dr. A. Monro at Edinburgh during the latter half of the last century. It was founded upon the incompressibility of the substance of the brain, and upon the spheroidal form of the skull. The idea might probably be traced from Monro to Boerhaave, or Pitcairn, or to some of their disciples. When, however, the last of the iatro-mathematical school had disappeared, we find Dr. Kellie taking up and defending this one of its doctrines which Monro had promulgated, and enlisting the late Dr. Abercrombie amongst *his* disciples. The name of Dr. Abercrombie was a tower of strength, and gave renewed vigour to the iatro-mathematical views. Dr. Clutterbuck adopted them in his article on Cerebral Apoplexy, in the 'Cyclopædia of Practical Medicine,' and even Dr. Watson, in his very recently delivered Lectures, maintained the invariableness of the quantity of blood in the brain, adducing the argument which Dr. Pitcairn, or even Borelli, would probably have advanced. "This depends," remarks Dr. Watson, "upon the mechanical structure of the cranium, and is capable of explanation upon the known principle of hydraulics." It is but justice to Dr. Watson, however, to observe, that in the second edition of his Lectures, he acknowledges a change of opinion on this point in consequence of Dr. Burrows's demonstration to the contrary.

The above history of this heresy, sketchy and slight as it is, shows that erroneous ideas reappear like weeds, and are as apt to perpetuate themselves in the soil of science. Dr. Burrows was impressed with the idea, that the opinions promulgated by Dr. Kellie and his successors were erroneous, and that they obscured the pathology of a most important organ; he therefore repeated the experiments of Dr. Kellie, and in the present work we have the results of his inquiries. These results, as well as the pathological views of Dr. Burrows, we now propose to place before our readers.

Dr. Kellie inferred from his experiments—1, that a state of bloodlessness is not discovered in the brains of animals which have died by hemorrhage, but, on the contrary, very commonly a state of venous cerebral congestion; 2, that the quantity of blood in the cerebral vessels is not affected by gravitation or posture of the head; 3, that congestion of the cerebral vessels is not found in those instances where it might be most expected, as in persons who die by hanging, strangulation, suffocation, &c.; 4, that, if there be repletion or depletion of one set of vessels (arteries or veins) in the cranium, there will be an opposite condition of the other set of vessels.

In examining these experiments analytically, Dr. Burrows clearly shows, that they do not bear out Dr. Kellie in the inferences he deduced from them, and that, in fact, Dr. Kellie did himself at first draw the inference that the brain of animals might be depleted by bleeding, and their vessels drained of a very sensible proportion of the blood contained in them. It was in a subsequent communication to the Medico-Chirurgical Society of Edinburgh that he contradicted himself. It appears that his experiments stand alone, and have not been repeated: Dr. Burrows's researches are therefore the more interesting. We subjoin the first *in extenso:*

"On the 11th of January, 1843, I killed two well-grown rabbits. The one (A, plate 1), by opening the jugular vein and carotid artery on one side of the throat; the other (B, plate 2) was strangled. Each animal died violently convulsed. A ligature was drawn tightly round the throat of the rabbit (A) immediately it expired, to prevent any further escape of blood from the vessels of the head. The rabbits were allowed to remain twenty-four hours on a table, resting on their sides.

"While the blood was flowing from the rabbit (A), the conjunctiva was observed to become pallid and the eyeballs to shrink within the sockets. Upon the examination of the head of this rabbit, the integuments and muscles appeared blanched and exsanguined. Upon removing the upper portions of the cranium, the membranes of the brain were found pallid, and scarcely the trace of a blood-vessel was to be detected on the surface of the brain. The longitudinal and lateral sinuses were nearly empty of blood, and their course was not denoted by any colour of blood. Upon making sections of the brain, the interior appeared equally exsanguined.

"Soon after the cord was drawn tight round the throat of the rabbit (B), the conjunctival vessels became congested, the eyeballs turgid, prominent, and even projecting beyond the margin of their sockets. The integuments and muscles of the head were found full of blood. Upon opening the cranium, the superficial vessels of the membranes, as well as the sinuses, were full of dark liquid blood. The whole substance of this brain and its membranes appeared of a dark reddish hue, as if stained by extravasated blood. The contrast between the two brains in point of vascularity, both on the surface and the interior, was most striking. In the one scarcely the trace of a blood-vessel was to be seen; in the other every vessel was turgid with blood." (p. 13.)

Dr. Burrows has appended coloured drawings of the post-mortem appearances in the brains of the two rabbits, and they are certainly in striking contrast. He observes, however, that the brains of sheep slaughtered by butchers are much less depleted than the brains of rabbits killed in the mode he describes. This difference is owing, he thinks, to a difference in the mode of death. Sheep, he observes, die partly from hemorrhage, and partly from division of the pneumogastric nerves, and of the cervical position of the cord.

Another contradiction to the iatro-mathematical doctrine regarding the cerebral circulation is contained in a tabular synopsis of the principal pathological appearances met with in 72 insane patients of Bethlem Hospital, examined by Mr. Lawrence, and published by Dr. Webster in the twenty-first volume of the 'Medico-Chirurgical Transactions.' In 53 of the 72 cases examined, the vessels of the brain were found congested; in one only were they exsanguine—"unusually empty"—and in this case death was immediately consequent upon the hemorrhage induced by the rupture of a femoral aneurism. Dr. Burrows, therefore, observes:

"Hence it is not a fallacy, as some suppose, to assert that bleeding diminishes the actual quantity of blood in the cerebral vessels. By abstraction of blood we not only diminish the momentum of blood in the cerebral arteries, and the quantity supplied to the brain in a given time, but we actually diminish the quantity of blood in those vessels. Whether the vacated space is replaced by serum, or resiliency of the cerebral substance under diminished pressure, is another question, into which I do not now enter." (p. 15.)

Can posture influence the cerebral circulation? We suspect each of our readers need only stoop to the ground to answer the question experimentally; yet the iatro-mathematical theory prevented the due reception of the results of daily experience, and apparently led Dr. Kellie into a wrong appreciation of some experiments he instituted on rabbits. As Dr. Burrows simply repeated these experiments of Dr. Kellie, we will subjoin his account:

"On the 28th of December, 1842, two full-grown rabbits were killed by prussic acid, and while their hearts were still pulsating, the one (C, plate 3) was suspended by the ears, the other (D, plate 4) by the hind legs. They were left suspended for twenty-four hours, and, before they were taken down for examination, a tight ligature was placed round the throat of each rabbit, to prevent, as effectually as was possible, any further flow of blood to or from the head, after they were removed from their respective positions.

"In the rabbit (C) the whole of the external parts of the head, the ears, the eyeballs, &c., were pallid and flaccid; the muscles of the scalp and bones of the cranium were also remarkably exsanguined. Upon opening the cranium the membranes and substance of the brain were pallid, the sinuses and other vessels were exsanguined; anæmic beyond my expectation.

"In the rabbit (D) the external parts of the head, ears, eyeballs, &c. were turgid, livid, and congested; the muscles and bones of the cranium were of a dark hue, and gorged with blood, which at some parts appeared extravasated. Upon opening the cranium, the membranes and vessels were dark, and turgid with liquid blood; the superficial veins were prominent, the longitudinal and lateral sinuses were gorged with dark blood, and there was staining of the tissues, if not extravasation of blood into the membranes. The substance of the brain was uniformly dark, and congested to a remarkable extent.

"Dr. Kellie asserts, but I think his experiments do not support him, that the contrast in the appearances within the heads of the two animals was but trifling. In my analogous experiments the contrast was most striking. In the one was to be seen a most complete state of anæmia of the internal as well as external parts of the cranium; in the other a most intense hyperæmia, or congestion, of the same parts; and these opposite conditions of the vascularity of the brain induced solely by posture, and the consequent gravitation of the blood.

"If the cranium were the perfect sphere, as taught by Monro, and as subsequently entertained by Abercrombie, and other distinguished writers on the pathology of the brain, these effects on its circulation (which I have now described) ought not to have resulted from the force of gravity on the blood in the cerebral vessels." (pp. 17-18.)

Four coloured plates illustrate this account; and certainly the contrast is as striking as in the experiments described previously.

These researches of Dr. Burrows ought, we think, to be considered conclusive on the question, so far as experiments of the kind can be considered conclusive.

It appears to us, however, that the original theory has had much more importance attached to it than it deserves. In the first place the cranial contents cannot be considered as free from atmospheric pressure any more than the heart, liver, &c. The cranium is *not* a perfect sphere, while the sinuses of the dura mater, situate within it, communicate with the exterior of the skull by several large veins. Then, if it be granted that the skull is a perfect sphere, and the amount of fluids must at all times be equal, the necessary equalization is attained by the pressure of the columns of blood forced in by the heart, which columns, dividing and subdividing until, at length, lost in the capillaries, exercise in the meanwhile a compressing power upon the contractile tunics of the vessels, and when this is overcome, upon the surrounding nervous tissue. This complicated structure of vessels and nervous fibrils cannot, we think, be properly compared with a homogeneous fluid, nor can the laws of hydraulics be applied to it. In short, we think that the cranium cannot be rightly likened to a beer-barrel, nor its contents to beer; nor do we think it necessarily follows that, because the brain is incompressible, its functions may not be destroyed by compression; indeed, we believe this is acknowledged by the spheroidal theorists. The pressure, they allow, may be so great as to rupture the vessels, whether that pressure be induced by retardation of the blood in the venous circulation, or by increased energy of the arterial circulation. That the quantity of *fluids* within the cranium generally cannot be sensibly increased, is probable nevertheless; but this is not, in truth, the prominent and fundamental proposition of the iatro-mathematicians. It was, really, that the quantity of *blood* could not be sensibly increased; the other proposition was only secondary to this. The one had a great therapeutic importance, because it bore on the propriety of bleeding and other points in the treatment of apoplexy, and was, therefore, adopted exclusively by those writers whose opinions Dr. Burrows combats; the other, having no practical value, was not noticed. But, although we believe the iatro-mathematical theory never received the full consent of the profession, or was generally acted upon in the treatment of congestive affections of the head, yet it threw an air of doubt and obscurity about an important point in physiology, which has materially tended to render the progress of cerebral pathology slow and imperfect. By the removal of a great error, Dr. Burrows has done as good service to the medical commonwealth as if he had discovered a great truth. We here, however, subjoin an important caution given by him:

"It may now be affirmed that the encephalon is not exempt from this law in physics—the gravitation of the fluids to the lowest parts of the corpse. The discovery of the operation of this force on the blood within the cranium after death suggests a precaution very essential to be followed, when it is desired to ascertain the precise amount of congestion of the cerebral vessels at the time of death. In such cases a ligature should be placed round the throat of the corpse, and drawn sufficiently tight to compress the cervical vessels, and arrest all flow of blood through them. This precaution will be most required in the examination of bodies where, from the kind of death, the blood may be suspected to remain fluid in the heart and great blood-vessels. The depending or elevated position of the head, during the examination of the body, will not then induce deceptive appearances, which mislead us in our conclusions as to the previous amount of congestion in the cerebral vessels." (p. 20.)

This caution is rendered the more necessary, because the non-congested state of the brain which has been observed in persons who have been hung, or strangled, has been adduced (vide Dr. Kellie's paper) in proof of the iatro-mathematical proposition. But Dr. Burrows shows that the brain may or may not be congested according to circumstances, and these circumstances consist in the mode in which the strangulation is effected, and in the anatomy of the cerebral vessels. He thus accounts for the occasional absence of cerebral congestion in those who have suffered death by hanging:

"When criminals are hung by the executioner, the knot of the rope is usually adjusted on one side of the neck; and it is found, after death, beneath the ear, resting on the mastoid process. It has been often observed, in the dissection of such criminals, that the cheek and integuments on this same side of the head are not near so livid and congested as on the other side. The pressure of the rope has not completely obstructed the return of blood through the external jugular vein on the one side, although it has effectually stopped the current on the other. In such cases it is probable that the deep-seated internal jugular vein on the one side has only been partially compressed, and has permitted to a certain extent the return of blood from the internal parts of the cranium.

"But there is another still more efficient cause of this occasional absence of congestion of the cerebral vessels after death by hanging: it is the subsidence of the *fluid* blood after death, while the body is yet suspended, through the cervical vessels which are not completely obliterated by the pressure of the cord. And it should be recollected that there are some channels which are scarcely, if at all, affected by the compression of the rope. These other channels are the vertebral sinuses and spinal plexus of veins, so ably delineated by M. Breschet." (pp. 25-6.)

The sinuses of the cranium may also be drained otherwise than through the vertebral sinuses. In examining the bodies of those who have died by strangulation, the great vessels of the neck are usually cut across to get at the thoracic viscera; and then, when the head is elevated to open the skull, the blood gravitates, and flows from the cut ends, and the blood-vessels (previously congested) are rendered comparatively empty. Dr. Burrows further observes, in proof of this explanation, that in other instances where life has been destroyed by obstruction of the respiration, congestion is usually present.

The cerebral arteries and veins were supposed by Dr. Kellie and his disciples to stand in such relation to each other that, although the quantity of blood in the one might be diminished, it would be proportionately increased in the other. In states of anæmia, for example, a diminished quantity of blood might pass into the cerebral arteries, but a proportionate increase would take place in the cerebral veins and sinuses; and so the whole volume of blood within the skull would remain the same. Now there is no proof that such a relative condition of the two classes of vessels exists. Dr. Burrows very ingeniously shows, too, that the extra-vascular serum contained in the encephalon plays an important part in equalizing the pressure and the contents of the cranium. This point is, however, discussed in the next section. We need only observe, further, that to strengthen his case Dr. Burrows adduces the recorded cases of pathologists to prove that the brain is liable to be congested, loaded with blood, &c.; so that the quantity of the latter is a variable quantity,

*On vascular pressure within the cranium.* After objecting to some theoretical views regarding the incompressibility of the brain, Dr. Burrows proceeds to observe that the force which is impressed on the cerebral substance, through the momentum of the blood in the cranial vessels, is derived partly from the left ventricle of the heart, and partly from the reflux of the venous blood during expiration. When the brain is exposed in the living animal, the dura mater is found to rise and fall a little alternately with every systole and diastole; and experiments show that this alternate rise and fall is really (as it appears to be) dependent upon cardiac action. As to the venous reflux, we quote Dr. Burrows's lucid statement:

"If the dura mater be exposed to view, and observed during the period of expiration, when the free return of venous blood from the brain is impeded, and a larger quantity of arterial blood is distributed with increased force towards that and other organs, the surface of the membrane is seen to rise; the brain itself swells and becomes turgid, but again subsides with the succeeding inspiration. All physiologists who have considered this subject agree in regarding these latter respiratory movements of the brain as partly, if not wholly, attributable to the reflux of the blood in the veins during expiration. Ecker, indeed, attributes these movements of the brain in great part to the ascent of the cerebro-spinal fluid during expiration. The last-mentioned writer has detailed numerous experiments, which show that if ligatures be placed upon the two carotid arteries of an animal, these cerebral movements accompanying respiration are not suspended; but if the external jugular veins in dogs (and these are the principal cerebral veins in these animals) be tied, these respiratory movements are much weakened, but not altogether destroyed; and if the cervical vessels, both arterial and venous, be divided, all movements of the vein are immediately lessened; and when the loss of blood becomes excessive, they cease altogether, and there follows a remarkable shrinking of the organ. But the effects of this reflux of the blood in the veins upon the cerebral substance are still more manifest after those accidents where, with loss of a portion of the bones of the cranium, there is also a laceration of the dura mater. In such cases the distending forces of the vessels acting on the cerebral substance are so strongly exhibited, that not only is the alternate rising and sinking of the exposed surface observed to correspond with each expiration and inspiration, but portions of the substance of the brain are actually protruded through the opening in the dura mater and bones of the cranium. Hernia of the convolutions of the brain is thus effected by a force from within, just as hernia of the convolutions of the intestines is produced by analogous forces, when the walls of the abdomen are accidentally perforated." (pp. 46-7.)

Such being the causes of cerebral pressure in continual action, it remains to ascertain what provisions have been made to equalise this pressure, so liable to vary greatly in amount, and acting on so delicate an organization as the brain, confined in its unyielding bony case. This is to be found in the cephalo-rachidian fluid. We have already, in our

Fourteenth Volume, given a *précis* of Majendie's views respecting this fluid. From experiments instituted by Ecker, we learn that the spinal theca rises and falls like the brain, concurrently with expiration and inspiration. If the theca be carefully divided, so as to leave the arachnoid membrane intact, the latter appears at the opening like a fine bladder containing fluid. This fluid is the cephalo-rachidian, and is manifestly subject to alternate increase and diminution of pressure, concurrently with the respiratory acts. It is engaged in a perpetual alternation of ascent and descent, and presses against the solid parietes with a force equal to the internal momentum. Dr. Burrows thus states his views of its functions :

"This extra-vascular serum appears to me to be supplemental to the other contents of the cranium; it is removeable by pressure or absorption ; at one time giving place to an increased quantity of blood in the cranium, at another, making up for a deficiency of blood in the vessels of the head. This extra-vascular serum not merely acts as supplemental to the varying quantity of blood, but also to the variable quantity of nervous matter in the brain. Its quantity is in the inverse proportion to the quantity of this nervous matter. Thus, in hypertrophy of the brain, there is a most remarkable deficiency of serum within the cranium ; the brain, its ventricles and membranes, are so devoid of this fluid, that they are almost dry; on the contrary, in atrophy of the organ, the ventricles and membranes are distended with fluid.

"This extra-vascular fluid may probably perform another office: perhaps through this cerebro-spinal fluid a more equable pressure is diffused over the whole mass of the brain and cord ; and, for the reception of this regulation of pressure, may be the contrivance of those cavities called ventricles, which dip into the central parts of the brain. The sac of the arachnoid may subserve the same purpose." (p. 56.)

This theory is plausible, and will certainly serve to explain some obscure points in cerebral pathology, but we apprehend its plausibility will prevent a ready adoption of it by pathologists, without corroboration by further experiments and observations. Essentially, it considers the spinal column and cranium as one cavity, and consequently reference must be had for the future to the functional condition of the spinal cord in all cases of cerebral compression. The amount of the cephalo-rachidian fluid must exercise, if this theory be correct, a most important influence on the functions of the cerebro-spinal axis. The means and agencies by which that amount may be diminished and increased, will also have an important bearing on the pathology and therapeutics of the brain and spinal cord. Dr. Burrows details a case of chronic inflammation of the investing membranes of the brain, with copious serous effusion, and which latter, in his opinion, by varying in site and amount, was the cause of a peculiar variation of the symptoms. The patient presented "a remarkable alternation between excitement and calm, between returning power and paralysis. On one day she had no command over the sphincters, and the lower limbs appeared almost powerless and insensible, but exhibiting slight involuntary movements when the feet were pricked. On other days she called for the bed-pan, and the legs were immediately drawn away with an expression of pain upon a slight amount of irritation." We think this case extremely inconclusive, since other causes might have been in operation, as, for example, recurring alternations of vascular action and atony.

It is certain, indeed, that the pathological influence of this fluid must always be difficult to appreciate, until its physiological relations and behaviour are better understood. There are some anomalous cases of paraplegia which may ultimately be elucidated by this theory; we allude to those examples in which there is no perceptible disease, either of the investments or structure of the spinal cord, and in which, upon some vivid emotion occurring, the paraplegia temporarily disappears. Now it *may* so happen (we put the case confessedly as a remote hypothesis), that in such examples there is a deficiency of pressure upon the dorso-spinal cord, because of a diminished quantity of the cerebro-spinal fluid, and that when in consequence of an emotion, the brain receives a greater influx of blood, the pressure then becomes sufficient for normal action;—that, in fact, the dorso-lumbar cord in such cases is permanently in a condition like that of the brain in syncope. The following are Dr. Burrows's general deductions:

"The foregoing inquiry will elucidate many interesting phenomena observed in states of vascular congestion or depletion within the cranium. When arterial or venous congestion of the brain is suddenly induced, the first effect will be an increased pressure on all the contents of the cranium; this pressure will cause the expulsion of a portion of this extra-vascular serum into the spinal canal. On the other hand, when abstraction of blood from the cranium is effected, there is a tendency to shrinking of the encephalon, but the vacated space is immediately occupied by a certain quantity of this serum. But when the power of the heart is inordinately increased for any length of time by stimulants, general plethora, or hypertrophy of the left ventricle, a train of symptoms is remarked similar to those produced by varying degrees of mathematical pressure artificially exerted on the brain.

"These effects of increased determination of blood to the brain may be witnessed even where there is no structural lesion of that organ, although the effects are probably partly obviated by the anatomical peculiarities just described; but they will be most strikingly displayed where there is pre-existing structural disease in the cranium.

"When obstruction to the return of blood from the brain takes place, so that the blood becomes almost stagnant in the sinuses, that part of the force of the left ventricle (which, in the normal state of the cerebral circulation, is expended partly in propelling the blood onward through the capillaries towards the right auricle, and partly in distending the vessels through the cranium) is, under such circumstances, expended upon the interior surface of the cerebral blood-vessels. This pressure is partly sustained by the resistance of the vascular tissues, and the remainder is borne by the surrounding cerebral substance. Whatever this force may be, it becomes a source of increased pressure upon the cerebral substance; and the more so, according to the pre-existing morbid states of the encephalon and its vessels.

"When the circulation is thus excited or obstructed, an obvious state of congestion of the integuments of the head and face is produced, and from the experiments I have detailed, it may, I think, be inferred that a simultaneous congestion of the internal vessels of the cranium is formed.

"In previously healthy conditions of the cranium, when it contains nothing but the brain and normal quantity of serous fluid, the cerebral substance may readily accommodate itself to a temporary increase of blood in its vessels (arterial or venous, or in both), and to the consequent pressure, by expulsion of a certain amount of serum; but when the cranium contains abnormal and unremoveable substances, then the brain cannot bear these accessions of vascular fulness and consequent pressure.

"In those pathological states of the encephalon where there is an increase in the quantity of solid matter within the cranium, and a diminution of the quantity of extra-vascular serum, as in hypertrophy of the brain, tumours and cysts in that organ, and in large extravasations of blood on the surface, every cause which is capable of exciting the heart's action produces a notably increased disturbance of the functions of the brain. The variable character of the symptoms of cerebral disturbance in these permanent lesions within the cranium, are thus probably accounted for by the variable vascular pressure. Andral has offered a nearly similar explanation of the occasional recurrence and intermittent character of cerebral symptoms, although their supposed cause, organic disease in the cranium, is permanent. It seems to me probable that many permanent structural lesions within the cranium do not affect the functions of the brain by pressure, except when there is some cause in operation capable of inducing vascular congestion, or when the lesion is of a mechanical nature, or is gradually increasing." (pp. 58-60.)

Dr. Burrows thinks it necessary to combat some views lately published, in which syncope is represented as being allied to apoplexy, and dependent on venous congestion. He maintains that it results from insufficient vascular pressure, and not from the inadequate supply of blood to the brain. In syncope arising from strong moral emotion, the quantity and quality of the blood, he argues, are unaltered previously to the attack, and the suspension of the cerebral functions arises solely from the diminished energy of the heart. We apprehend that these views of Dr. Burrows will not meet with more general acceptance than those he combats. It may be justly argued, we think, that if the heart be so enfeebled as not to be able to propel the column of blood with the force necessary to cerebral action, it will propel a column of diminished size, or in other words, in syncope, the heart's action being enfeebled, the contractility of the arteries will react upon the column of blood, and by rendering it smaller, diminish the supply of blood to the brain, as is generally supposed. Now the importance of a due supply of blood is, we think, sufficiently manifest from the great vascularity of the gray substance. Dr. Burrows argues, that in cases of hypertrophy of the brain, the substance of the organ is extremely anæmic, for the hypertrophy, by filling the skull, completely prevents the blood entering the capillaries. Dr. Burrows cannot wish his readers to understand that the *quality* of the blood has not quite as much influence on the functions of the brain as the quantity. In cases of anæmia from hemorrhage or defective sanguification, it does not appear probable that the amount of the fluid circulating through the vascular system is diminished, nor is it improbable that the increased rapidity of the heart's action in a great degree counterbalances the diminished force of the systole. Besides, we have examples of cardiac affection, unaccompanied by an anæmic condition of the blood (meaning by anæmic, a deficiency of the red globules), in which the systole is slow and feeble, and yet there are few of the characteristic symptoms of anæmia. There can be no doubt that the horizontal position increases the vascular pressure on the brain, when the heart's action is enfeebled, but it also increases the quantity of blood passed through the brain in a given time. We would ask also, if we grant (as we willingly do) that a certain amount of vascular pressure is favorable to the functional activity of the brain, how is it that this acts? Is it not by favoring the circulation through the numerous capillaries, and thus increasing the assimilating activity of the cerebral substance? And, if so, may not the proper condition of the matter to be assimilated (the blood) have as important an influence on the organ as the proper condition of the vessels that convey it, or the organs that assimilate it? We think when Dr. Burrows inquires how it is that diminished vascular pressure interrupts cerebral function, he will be induced to modify his views in some degree.

*Cerebral disorders from ligature of the carotids.* It has long been known that compression or ligature of the carotid arteries has an important influence upon the condition of the brain. A female was operated upon by Mr. Key for aneurism of the inominata.

"In about an hour and a half after the operation she appeared asleep, the respiration being natural, with the exception of a snore. This noise gradually became fainter, and she expired in about two hours more; just four hours after the operation. Upon examining the body it was found that the opening of the other (left) carotid from the arch of the aorta was nearly closed; it was scarcely one tenth its natural size. The substance of the brain was healthy; its vessels sound, and containing the ordinary quantity of blood. There was a little serous effusion between the membranes."

Now, Dr. Burrows (a little influenced by his *idolum specûs,* we think) observes that "there appears nothing to account for this woman's death but the sudden diminution of the momentum of the blood in the cerebral arteries, occasioned by the closure of the only pervious carotid." But surely a defective *supply* of blood to the brain, in this case, so much below the ordinary quantity sent to it, may be allowed to go for something, and that the want of blood, as well as the want of pressure, was a cause of death. In cases in which one carotid has been tied, the cerebral disorder has been characterized by hemiplegia of the side opposite to the obstructed artery, with disorganization of the hemisphere of the same side. These results may, in part at least, be very fairly attributed to defective nutrition of the hemisphere implicated; but Dr. Burrows, faithful to his theory, says that the cause of the commencing hemiplegia is the pressure induced on it by the vascular distension of the opposite hemisphere. There is no proof, however, that one hemisphere can thus compress the other; but, on the contrary, it is probable that the falx cerebri will effectually prevent such a result, just as the tentorium protects the cerebellum from compression by the cerebrum.

Considering that compression and ligature of the carotids have been found useful in epilepsy, mania, and other chief-diseases, Dr. Burrows thinks that the ligature may be resorted to in hopeless cases of the kind alluded to.

"Although the ligature of the common carotid is attended with risk to life in some cases (perhaps in the proportion of one death in four operations), still experience proves that where proper precautions have been taken the operation is not so dangerous as many suppose. Therefore, in violent and hopeless cases of epilepsy and some kindred maladies, which are characterised by extreme cerebral congestion, it appears to me that, other remedies failing, this operation may be fairly resorted to. I am aware of the responsibility of advocating a remedy attended with risk to life; but are not all our best remedies most violent poisons in the hands of the unskilful? But this truth does not forbid their use by the more expert. So may this powerful method of influencing the cerebral circulation be justifiable in aggravated cases of the class referred to, and where the precept of Celsus, '*satius est anceps remedium experiri quàm nullum,*' may be fairly put into practice." (pp. 78-9.)

We would only observe that there is another good precept to be remembered, and that is "Saltem non nocere."

*Causes of coma.* The progress of neurology, during late years, is amply marked by the clearer comprehension of the phenomena of cerebral diseases. What is the condition of the brain that produces coma? was a question not easily answered twenty years ago; and the answer involved the hypothesis of an obstruction to the flow of the "nervous fluid," or a confession of ignorance. The difficulty of explaining this condition was increased by the circumstance that often no appreciable change in the constitution, or vascular system of the brain, could be detected after death from profound apoplexy. Dr. Abercrombie has particularly noticed examples in which not even congestion of the brain was observed. Dr. Burrows's explanation is, that the active depletion practised before death, in two of the cases mentioned, had emptied the cerebral vessels, and that the apoplexy was immediately dependent on sudden vascular pressure. Now although bloodletting may relieve this, the respiration has already become involuntary, slow, and stertorous, and the blood decarbonised; so that the coma is kept up by the circulation of the latter through the brain, and the patient actually dies of slow suffocation. It is probable that in deaths from acute laryngitis, when the respiration has been restored by tracheotomy, and in deaths after resuscitation from hanging, drowning, and suffocation from irrespirable gases, there is the same fatal condition of the brain.

Is the coma in the so-called serous apoplexy dependent on pressure from serous effusion, or on vascular pressure from congestion? Dr. Burrows argues in favour of the latter. He thinks the diagnostic symptoms between serous and sanguineous apoplexy are more fanciful than real; that, in fact, the serous effusion is itself a sequela of vascular congestion, and that the serous apoplexy is no other than simple apoplexy, that is to say, cerebral congestion terminating in effusion.

From our review of Dr. Neisser's work (see the preceding article), it will, we think, be clearly manifest that serous effusion is one of the modes in which the subacute form of arachnitis terminates. In such a form of arachnitis there is doubtless vascular congestion; but we cannot conclude with Dr. Burrows that that congestion is the cause of the coma and other signs of a fatal termination. We have no evidence of "vascular pressure" during the earlier stages of the disease, when the vascular action is the greatest. On the contrary, there is an increased activity of the cerebral functions. It is only when the serum accumulates within the cranium, and especially at the base of the brain, that the comatose symptoms come on (vide p. 405); for it must be remembered that the seat of the effusion has an important influence on the development of symptoms of pressure.

Dr. Burrows applies similar views to extravasation, and advances that the effused blood is not the cause of the apoplectic coma. We will quote his opinions on this point:

"I am of opinion that apoplectic coma is rarely dependent upon the extravasation of blood, although the concomitant paralysis undoubtedly is. Upon the examination of the brains of apoplectic patients we sometimes find large extravasations of blood, which, from peculiar appearances in the clot, we feel assured have existed there for many days, or even weeks, and yet during the greater part of that period there has been no coma. Upon other occasions we discover small extravasations of blood within the brain, which, from their appearance, we can decide have only been effused a short time prior to death; and, nevertheless, there has been a well-marked coma in these cases. Hence, if pressure be regarded as the physical cause of apoplectic coma, and that pressure is supposed to be occasioned by the extravasated blood, then we must account for the paradox of a small extravasation producing a coma which terminates fatally, and a large effusion of blood having no such effect." (p. 92.)

It appears to us that Dr. Burrows is wanting in his usual lucidity and precision here. There appears somewhat of special pleading in the argument. The observation we have just made respecting the importance of referring to the exact locality of the extra-vascular causes of pressure applies with peculiar force to extravasation of blood. In the two cases related by Dr. Burrows in proof of his views, the influence of a hemorrhagic site is very clearly shown. We have already observed in the article referred to that the effects of pressure from extra-vascular causes are manifest in proportion as the locality of the compressing cause is near the base of the brain. Now in the case of Mrs. G. (detailed by Dr. Burrows) the first extravasation of blood was "a large clot, probably an ounce, effused into the anterior half of each anterior lobe;" and the symptoms when this occurred are thus described:

"In the forenoon of Sept. 12th she had complained of vertigo and drowsiness while engaged in her domestic affairs. She did not fall or lose her consciousness, but contrived to get up stairs to her bed-room. She was assisted to her bed, where she remained perfectly sensible for some hours, but stupor increased; so that, towards evening, she could not be roused at all. Her family now became alarmed, and sent for medical assistance. She was found lying on her back, perfectly insensible; her face pale, and expression placid; hands cold; breathing natural; pulse 120, small, and feeble; pupils dilated, and insensible to the influence of light."

On the following day she was restless and delirious; so much so, indeed, that she could hardly be kept in bed—a state of things quite unlike apoplectic coma. The second extravasation, occurring about forty-eight hours before death, was beneath the cerebral arachnoid over the left hemisphere, and at the base of the brain; all the ventricles, although not distended, contained clots of soft and uniformly black blood. There was also a considerable quantity of blood effused into the tissue of the spinal pia mater, especially over the medulla portion. This blood, Dr. Burrows thinks, was probably derived from the ventricles of the brain, it having followed the course of the cephalo-rachidian fluid. The symptoms accompanying this extravasation are thus described: "Her countenance was haggard; her eyes fixed and vacant; she was roused with difficulty; her articulation imperfect, and she answered incoherently. She gradually became comatose, and sank on the third day after her admission." Here we have a state of things very different to the preceding, with a corresponding difference in the seat of the extravasation. Of course this solitary case would prove nothing; but as it is corroborated by numerous similar cases, we must fairly presume that the difference in the seat of the effusion was connected with the difference in the symptoms.

The next case is equally instructive. A female had a series of fits. The last and fatal fit was profoundly apoplectic, with coma, stertor, and death in twelve hours. On examining the corpse there was a clot of recently effused, dark-coloured blood, extending nearly across the tuber annulare. The penultimate fit (six weeks previously to the fatal attack) was complicated with hemiplegia of the right (?) side. The corresponding post-mortem appearance was a diffused yellow staining in the substance of the right hemisphere, becoming deeper downwards, and a large and somewhat hard clot of blood external to the lateral ventricle, a softening or breaking down of the corresponding corpus striatum, and part of the optic thalamus.

The extravasation may also influence the development of coma, not simply by its immediate relation to important structures, but by its relation to the cephalo-rachidian-fluid. The pressure of extravasation spread over the hemispheres will be expended upon the whole depth of the brain before it can act upon the base, and, in particular, the cephalo-rachidian-fluid in the ventricles will be interposed like a water-cushion between the two surfaces. Should the extravasation be gradual, a corresponding absorption of this fluid will tend to equalize the pressure, and the symptoms may be comparatively trifling; for it is well known how extensively the hemispheres may be injured without serious consequences. But if the blood be extravasated at the base, it is in immediate and direct contact with the great trunk conductors to and from the seat of perception and intellect, and, by compressing them, or otherwise interrupting their functions, will interrupt the functions of the superincumbent mass.

*The connexion between apoplexy and diseases of the heart.* Having arrived at sounder physiological views, Dr. Burrows next proceeds to apply them to pathology. The state of the circulation through the brain must necessarily be influenced by all disorders of the heart—of that central force-pump by which the circulation throughout the system is maintained. Portal was the first writer who devoted any considerable attention to the connexion between apoplexy and cardiac diseases. Dr. Kellie's experiments and inferences probably prevented Portal's doctrines taking that root in the professional mind which might have been expected; and the omission of Dr. Abercrombie to note specially the action of a diseased heart on the cerebral circulation, in his investigations into the pathology of apoplectic affections, tended to keep them still further in the background. But if error multiplies itself, truth is never lost; and Portal's views began at last to modify the views of English pathologists. Of late years we think the doctrines as to the influence of cardiac disease on the vascular system within the cranium have been much less undecided than Dr. Burrows seems to suppose. Dr. Kellie's doctrines never had an extensive influence in *practice;* and so soon as Bright, Craigie, Brichteau, Bouillaud, Andral, Hope, &c., took up its pathology, the influence of cardiac disease in predisposing at least to apoplexy was clearly established. "It is found, for instance," Dr. Craigie observes, "that in most cases of apoplectic seizure, either the left ventricle of the heart is affected with hypertrophy or dilatation, or the mitral valve is ossified, and its aperture contracted; or the aortic valves are more or less diseased; or even the whole of the arch and part of the descending aorta may be ossified, roughened internally, and incapable of transmitting the blood properly." * Nevertheless, it is interesting to find that Dr. Burrows presents us with more precise data, founded upon tabulation by the numerical method. In the subjoined table he gives an analysis of 132 cases of apoplexy and sudden hemiplegia, derived from trustworthy sources, arranged with reference to the coexistence of cardiac disease:

| " Authors. | Cases. | Diseased Heart. | Per Cent. |
|---|---|---|---|
| Andral . . . . | 25 | 15 | 60· |
| Clendinning . . . . | 28 | 15 | 53·5 |
| Hope . . . . | 39 | 27 | 69·4 |
| Burrows . . . . | 34 | 23 | 67·6 |
| Guillemin . . . . | 6 | 4 | 66·6 |
| Total . . . | 132 | 84 | 63·6 |

"The inference from the foregoing calculation is, that in any given number of cases of apoplexy and sudden hemiplegia, no less than three fifths will present unequivocal signs of cardiac disease: either hypertrophy, dilatation, valvular disease, or some combination of these lesions. This proportion proves that the frequency is much greater than is commonly supposed, even by those who admit the occasional influence of cardiac disease in the production of apoplexy and hemiplegia." (p. 117.)

The order in which the pathological phenomena arise in cases of this kind is very neatly and lucidly stated by Dr. Burrows. The constantly augmented force with which the blood is projected from the hypertrophied left ventricle gradually dilates the cerebral arteries, and diminishes their contractile power. The surrounding cerebral substance thus becomes more liable to pressure from the column of blood sent through them, and on any sudden increase of this pressure coma may take place, or the tunic of an artery yield, and hemorrhage occur. Should there be an atheromatous condition of the arterial tunics, the latter is the more likely to take place. Valvular diseases of the left side of the heart act on the brain by exciting congestion, first of the pulmonary vessels, then of the right cavities of the heart, and, next, of the venous system within the cranium. While the blood is thus retained on the one side, if there be also hypertrophy of the left ventricle, it is propelled with increased force on the other; and thus the brain is included between two opposing forces, and both classes of vessels distended and dilated.

" The relative frequency of these several cardiac lesions in cases of apoplexy and sudden hemiplegia may be estimated from the following analysis of twenty-five cases recorded by Andral, and thirty-four cases taken from my own case-books:

| | No. of Cases. | Heart diseased. | Hypertrophy with valvular disease. | Hypertrophy (simple). | Valvular disease. |
|---|---|---|---|---|---|
| Andral . . . | 25 | 15 | 9 | 4 | 2 |
| Burrows . . | 34 | 23† | 10 | 6 | 6 |
| Total . . | 59 | 38 | 19 | 10 | 8 |

(p. 123.)

*Influence of age on apoplexy.* In considering cardiac disease as a cause

* Elements of the Practice of Physic, 1840, vol. ii, p. 885. See also the Edin. Med. and Surg. Journ., vol. xix.

† In one case there was simple dilatation of the cavities.

of apoplexy and hemiplegia, we have followed our author hitherto through his researches exclusively devoted to a consideration of the force-pump. But the condition of the conducting tubes merit, we think, an equally decided notice. On this point Dr. Burrows's work is defective, as he only incidentally alludes to the condition of the cerebral arteries in cases of apoplexy. It has occurred to every practitioner to observe that persons suffer from extensive disease of the heart, and yet very rarely experience an apoplectic attack, or any important symptoms which can be referred to the brain; and, upon further consideration, they will probably remember that almost all the cases of prolonged cardiac disease, thus free from cerebral complication, have occurred in young persons. We know of no numerical data which show the number of apoplexies in persons under twenty years of age; but it is remarkable that they are so few as to be thought unworthy of notice by Dr. Burrows and other numerical writers. In an elaborate table which Dr. Burrows has drawn up of the ages at which 215 cases of apoplexy and hemiplegia occurred, the cases being related from his own records, or from those of trustworthy observers, there is not one entered under the age of twenty. It is a circumstance worthy notice, too, that the numbers at each age are progressively greater as the age advances. We subjoin the very valuable table which Dr. Burrows has made; in which the proportion to the population living at each age is indicated—a most important estimate:

| Age. | No. of Cases. | Population of this age. | Proportion of cases in 1000 persons. |
|---|---|---|---|
| 20 to 30 | 16 | 3,000 | 5·3 |
| 30 to 40 | 30 | 2,500 | 12·0 |
| 40 to 50 | 40 | 1,800 | 22·2 |
| 50 to 60 | 41 | 1,300 | 31·5 |
| 60 to 70 | 54 | 1,000 | 54·0 |
| 70 to 80 | 30 | 500 | 60·0 |
| 80 and upwards | 4 | 200 | |
| Total | 215 | 10,300 | |

"It must be remembered that the figures in the second column of the preceding table do not represent the *actual* numbers of cases of apoplexy and hemiplegia occurring at successive ages in any given population (ex. gr. 20,000), but only the *relative proportion* of cases in each successive decade, and this is compared with the numbers living of the same age. The population is assumed to be 20,000, of whom about one half will have attained the age of twenty years; and the numbers living in the successive decennial periods will be *nearly* those assigned in the third column of the table." (p. 131.)

Dr. Burrows, adopting some views of Dr. Clendenning as to the increase in size and force of the heart, with increasing age, attributes this greater frequency of apoplectic affections at the more advanced ages to the increased force and size. Bizot found that the walls of the left ventricle, and the septum, increase in thickness as age advances; but he found, also, that a gradual change in the arterial tunics was equally constant. Baillie, Stevens, &c., have remarked how constantly they are affected in old age by atheromatous degeneration, and, indeed, considered it almost a normal state. Bizot found opacity of the membraneous portion of the semilunar valves in 80 per cent. of females aged from 60 to 89 years; and in 92 per cent. of men of the same age. The first traces of this change are usually observed in men about the age of 49. Considering, then, the constancy of atheromatous degeneration in persons aged above 50, we should feel inclined to think that the great predisposing cause of apoplexy at ages above that is this brittle condition of the arteries, whereby they are rendered unable to resist—not the column of blood impelled ordinarily by the hypertrophied left ventricle, but the accidental propulsion, or retention, of a larger quantity of blood than usual. In short, that the adaptive power existing in the resiliency of the arteries of youth and manhood is impaired in proportion as age advances; and that, consequently, in cases of apoplexy, we should look as carefully to the condition of the conducting tubes—the cerebral vessels, as to that of the central force-pump—the heart. Whether, however, the predisposing cause be in the one part of the circulating system, or in the other, or whether it be in both, the quotation from Goldsmith, by which Dr. Burrows illustrates the most suitable treatment, is not less apt:

> "To husband out life's taper at the close,
> And keep the frame from wasting by *repose*."

*Treatment of Apoplexy.* The practical deductions as to treatment, as given by Dr. Burrows, seem to us to be eminently sound and valuable, so far as they go. In the first place, the posture of the patient should be attended to; he should be so placed that the blood may gravitate from the head. Secondly, however valuable the indication by the pulse may be as to the propriety of bleeding, that operation should not be decided upon until the condition of the heart has been ascertained. Without this knowledge the pulse cannot be taken as a safe guide.

"But suppose a careful examination of the apoplectic or hemiplegic patient's heart discloses the existence of valvular disease, to the extent of obstructing the circulation through its cavities, here the pulse will be a most deceptive guide as to the propriety or impropriety of abstraction of blood. If the mitral valve be principally implicated, and allow of regurgitation from the left ventricle, the small and irregular pulse so commonly observed with that lesion would probably dissuade from that free abstraction of blood which the cerebral symptoms might require. If, in another case of apoplexy or hemiplegia, the aortic valves be found diseased to the extent of not only obstructing the onward current of blood, but also of allowing regurgitation into the ventricles, during its diastole, there will probably be associated with this lesion considerable hypertrophy of the left ventricle. Here will be observed a full and vibrating or thrilling pulse, but a pulse of increased action without real power, and hence a deceptive pulse; and one which, if it be regarded without reference to the structural changes of the heart, would invite to a more copious abstraction of blood than was called for by the general symptoms. In each of these last-mentioned cases, greater relief to the symptoms will be obtained by a free local abstraction of blood from the vicinity of the heart (either by cupping from beneath the left mamma, or between the left scapula and spine,) than by a much larger depletion by venesection." (p. 142.)

When the inflammatory stage supervenes, the symptoms

"May generally be controlled in a most striking manner by small local depletion from the temple or mastoid process on the side opposite to the paralysis; by the application of cold to the head, and by the administration of purgatives, by restricted diet, and by extreme quiet in the sick-room. In addition to these remedies I have found, when the heat of head is diminished, that a blister applied near the occiput affords great relief to the oppressive headache. If the patient be not very advanced in years, or extremely exhausted by depletion, great benefit will be derived at this stage of apoplexy from the administration of small doses of mercury. One grain of calomel may be given every six hours, leaving the mineral to act as a purgative, or to slightly affect the gums, but not allowing the mercury to produce ptyalism, which is generally very distressing to the hemiplegic patient, whose powers of mastication and deglutition are already impaired by his disease." (p. 146.)

The sequelæ of an apoplectic fit require great attention. The pains in the palsied extremity, dependent most probably on centric irritation, are only relieved by time. The changes in the brain gradually advance, and the affected parts at length put on a more healthy action. It is during the convalescent stage that the greatest circumspection is required, as patients are apt to resume their mental and bodily activity at a very early stage. If this be permitted, Dr. Burrows observes—

"One of three accidents may happen. First, if there be too early excitement of the cerebral circulation after the fit, and before the fluid parts of the extravasated blood are absorbed, and while the cerebral substance around is still soft from infiltration, and before a cyst is formed around the clot, a fresh extravasation will take place, and produce irreparable mischief.

"A second accident likely to ensue from too early exertion after apoplexy, followed or not by palsy, is a renewal of the head symptoms, and subsequent disorganization of the cerebral substance.

"A third serious consequence of a too early return to mental occupation, or the anxieties of business, is the supervention of inflammation of the brain and its membranes." (p. 149.)

So far as the circulatory system is involved, in the treatment of apoplexy, this view will, we think, be acknowledged to be extremely judicious. The treatment, however, of this class of affections involves more important and recondite points than the dynamics of a diseased heart. The circulation through the brain is liable to be impeded by numerous other agents: Diseases of the air-passages, lungs, and liver are amongst those which act like cardiac disease. Then there is that whole class of affections in which there is morbid change in the circulating fluid, as in disease of the kidneys; and another numerous class may be found in diseases of organs which exercise an incident excitor action on the brain. Gastric disorders belong to this category. Nor can arthritic affections, so frequently seen in men of a sanguineous temperament, and so constantly connected with atheromatous changes in the arterial tissues, be overlooked.

*The connexion between cardiac diseases and functional disorders of the brain.* It is a necessary conclusion, from the foregoing experiments and deductions, that diseases of the heart must have important etiological relations to functional diseases of the brain. There result from the disturbance of the cerebral circulation, vertiginous affections, cephalæa, nervous irritability; and even insanity, may (with a suitable concurrence of circumstances) be immediately dependent on cardiac disease. Dr. Burrows first relates five instructive cases, to show the influence of the latter in inducing epistaxis. In one of these the hemorrhage appeared to be periodic, and the whole warn us, that it should not be regarded as an isolated or unimportant symptom. Dr. Burrows even states his opinion that it may "often be considered as strictly pathognomonic of an obstructed circulation through the heart, as hemoptysis is symptomatic of tuberculated lungs, or intestinal hemorrhage of an indurated liver." Probably that congestion of the Schneiderian membrane, which excites sternutatio—one of the apoplectic molimina—is sometimes dependent on the same causes as the epistaxis.

*Headache.* This is often a prominent symptom in hypertrophy of the heart, and its origin is often mistaken.

"These persons generally have pallid faces, and peculiarly hard, incompressible pulses; they are troubled with severe and obstinate headache, vertigo, some impediment in the speech, muscular tremors, or imperfect palsy. The histories of these patients almost invariably agree in one particular—that they have been addicted to spirit-drinking; they are also liable to be attacked with profuse hemorrhages, and ultimately become affected with general dropsy. My attention was first directed to these patients by Dr. Latham, and numerous instances have I since seen; and the more closely I have studied them, the more frequently have I found such persons labouring under hypertrophy of the heart, without evidence of great valvular obstruction, but perhaps combined with albuminous urine." (p. 176.)

*Nervous irritability* is another affection consequent on functional disturbance of the brain, the origin of which may be traced to cardiac disease.

"Middle-aged persons, of both sexes, in the upper ranks of society, apply occasionally for medical assistance, and who are suffering from uneasy sensations in the head, lowness of spirits, feelings of debility, occasional faintness, disposition to sigh, urgent desire for fresh air about them, irritability of temper, incapability of steady occupation, disturbed rest at nights. Such persons are often supposed to be dyspeptic, hysterical, nervous, or on the verge of insanity. Neither they themselves, nor their ordinary medical attendant, have suspected the existence of any disease of the heart. Auscultation in these cases has several times revealed to me the physical signs of valvular disease in the heart, or of serious changes in the aorta, and then, upon more close inquiry, other symptoms indicative of interruption to the course of the blood through the heart are confessed to.

"The history of such individuals often informs us that they have suffered from rheumatic fever many years before, or they have had some previous severe inflammatory affection of the chest, most commonly pleurisy; or they remember, under the influence of mental excitement or bodily exertion, many years ago, having felt some peculiar sensation in the cardiac region, or that they suddenly fainted. The subsequent progress of such develops many more unequivocal symptoms of confirmed cardiac disease." (pp. 178-9.)

Dr. Burrows thinks if auscultation were generally employed among insane patients that numbers would be found to be suffering from cardiac lesions. Dr. Burrows rides his hobby pretty vigorously here. May not disease of the brain induce disease of the heart? We must not suppose the brain to be a passive organ; only acted upon, and never reacting. Cardiac diseases are certainly more frequently fatal amongst the insane than amongst the general population. In an interesting table given by Dr. Thurnam, in his 'Statistics of Insanity,' the deaths from diseases of the heart in the Retreat, near York, are 6·402 per cent. of the total deaths; while in England and Wales, according to the returns of the Registrar-General, they were only 1·075 per cent.

*The connexion between acute cardiac disease and cerebro-spinal affections.* Dr. Burrows gives a short literary history of the progress of our knowledge as to the connexion between acute diseases of the heart and spasmodic and maniacal affections. Six of these anomalous and deceptive cases have come under his own observation; so that he is led to believe they are of more frequent occurrence than is generally supposed. These and other examples from authors are detailed, the whole numbering sixteen cases of pericarditis, complicated with disease of the brain and spinal cord. Eleven of these were fatal; and Dr. Burrows observes, in not one of them could a trace of disease be discovered in the brain or its membranes, and a morbid change in the spinal cord in only two. In seven of the sixteen there was no rheumatic affection. In four of the cases treated successfully the diagnosis of cardiac disease was satisfactorily established; in the fifth it was suspected. In two of the eleven fatal cases cardiac disease was detected during life; in one it was suspected; and in the remaining eight was only discovered on examination of the corpse.

Various explanations have been attempted of the pathological connexion between functional disorder of the cerebro-spinal axis and cardiac lesions. Dr. Burrows adopts the explanation of Dr. Bright (which also closely coincides with that of Bouillaud); namely, that the inflammatory affection in these cases implicates the phrenic nerve, and that it is the more immediate means of communicating the irritation to the brain and spinal cord. Dr. Hope considered that the pneumogastric nerves might be the channel, while Dr. Watson is of opinion that the cerebral symptoms depend upon a disturbance of the cerebral circulation.

We are inclined to think, on a careful perusal of Dr. Burrows's cases, that they are too heterogeneous to be tabulated, or to form a basis for numerical deductions. The rheumatic cases should have been arranged in one division; for there is no analogy between case 1, headed "Active Articular Rheumatism, complicated with Carditis and Pericarditis, presenting the ordinary symptoms of an Inflammatory Affection of the Brain," occurring in one of the boys at Christ's Hospital, or case 13, of a young man labouring under general dropsy, and dying of apoplexy, and case 16, of a middle-aged man suffering from asthma and an extraordinary curvature of the spine. A careful analysis of such cases would, we think, show that our estimate of their value is just. In case 2 (related by Dr. Burrows) the head was not examined. In case 3, by Dr. Abercrombie, chorea and hysterical (?) delirium came on subsequently to the inflammatory attack, and were probably the result of excessive depletion; in this case, also, it does not appear that the head was examined. In cases 9 and 10, the supervention of dementia and insanity was a sufficient proof of morbid change in the brain or membranes.

We think that the cerebral symptoms in cases of the kind described by Dr. Burrows do not arise from one or two causes only. When it can be distinctly shown that the inflammatory action in the pericardium has implicated the phrenic nerve, the theory of Bright and Bouillaud, adopted by Dr. Burrows, will suffice. But Dr. Burrows should not, we think, have omitted to point out distinctly the cases in which this nerve *was* implicated. In six of the cases we might *suppose* such a complication, in the remainder there is not even ground for the supposition. On the other hand, it must be remembered how many cases of pericarditis occur without this cerebro-spinal complication.

Another cause operating in the rheumatic forms of this class of cases, is a metastasis of the morbid action to the membranes of the brain and spinal cord. Dr. Burrows, without denying its occasional occurrence, objects, that in not one of the eleven cases could a trace of disease be discovered in the brain and its membranes. Dr. Burrows has himself explained how excessive congestion may exist before death, and yet be undiscovered after death. But we apprehend that it is precisely in rheumatic metastases we might reasonably expect such a result, without allowing for the effects of depletion. We see rapid metastasis from one joint to another, inducing apparently violent inflammatory action and impeding the functions of the articulation, and yet leaving no trace of its previous presence after it has disappeared. Just such a state of things may occur to the serous membrane of the brain. Further, changes may be thus induced in the delicate structure of the brain, which, inappreciable to the naked eye, would be rendered obvious by the microscope, and yet no mark of abnormal vascular action remain.

In chronic cases, the complication of renal disease with pericarditis is not unfrequent; and, as one of the principal secondary results of structural disease of the kidneys is to disorder the functions of the brain, as well as of the serous membranes generally, we think attention to those organs in cases of the kind under discussion is essentially requisite, if we would attain a perfect knowledge of their pathology. Now, we do not find that Dr. Burrows has directed his attention at all to this important point. Lastly, we think vascular pressure from the diseased heart sufficient under a suitable combination of circumstances to develope the complicating symptoms.

*Treatment.* Although we have differed in opinion with Dr. Burrows as to the pathology, we fully coincide with him in enforcing the necessity of an early examination of the heart by means of auscultation in all obscure and intractable affections of the brain and spinal cord. Dr. Burrows's plan of treatment is, to deplete repeatedly from the region of the heart by cupping-glasses applied between the left scapula and the spine, or by the application of leeches near the left mamma. Venesection may be indicated in robust persons, and he is not of opinion that free depletion from the arm favours a metastasis to the heart in rheumatic inflammations. As regards counter-irritants,

"There is no remedy," Dr. Burrows observes, "upon which, after local depletion, I place more reliance, in the treatment of pericarditis than a blister, and, in the more severe cases of pericarditis, characterized by hurried respiration, anxious countenance, great dyspnea, short cough, frequent small pulse, with the physical signs of pericardial effusion, the amendment consequent on the application of a large blister to the sternum is so rapid and striking, that it can hardly be realized by those who are not in the habit of contemplating such cases.

"In all cases of pericarditis, excepting in patients affected with tubercular disease of the lungs, or in the very anæmic and cachectic, I employ mercurial preparations as freely as in acute inflammations of other parts."

He administers

"A few large doses of calomel, varying from five to ten grains, combined with a grain of opium, and then continue half the dose of the same combination at moderate intervals, until the urgent symptoms abate, or the mouth becomes distinctly affected by the mercury."

Dr. Burrows would have written a more valuable monograph, had he cultivated a more extensive acquaintance with recent authors, both British and foreign. It is rather late in the day to quote a writer like Dr. Cooke. It is clear, too, that Dr. Burrows is warmly devoted to his *idolum specûs;* so manifest, indeed, that no harm we are sure will result from the great prominence he gives to the etiological relations of cardiac disease in affections of the brain. The work, however, taken as a whole, is excellent; it has the characteristics of an English production in an eminent degree; it is lucid, precise, practical; and will, we think, establish Dr. Burrows's reputation.

---

